# Coexistence of congenital left ventricular aneurysm and prominent left ventricular trabeculation in a patient with *LDB3* mutation: a case report

**DOI:** 10.1186/s13256-017-1405-1

**Published:** 2017-08-19

**Authors:** Shengshuai Shan, Xiaoxiao He, Lin He, Min Wang, Chengyun Liu

**Affiliations:** 1 Department of Geriatrics, Union Hospital, Tongji Medical College, Huazhong University of Science and Technology, Wuhan , 430022 China; 2 Department of Ultrasound, Union Hospital, Tongji Medical College, Huazhong University of Science and Technology, Wuhan , 430022 China; 3 Key Laboratory of Molecular Targeted Therapies of the Ministry of Education, Institution of Cardiology, Union Hospital, Tongji Medical College, Huazhong University of Science and Technology, Wuhan, 430022 China; 4 The First People’s Hospital of Jiangxia District Wuhan City & Union Jiangnan Hospital, HUST, Wuhan, 430200 China

**Keywords:** Congenital left ventricular aneurysm, Trabeculation, *LDB3*, Mutation

## Abstract

**Background:**

The coexistence of congenital left ventricular aneurysm and abnormal cardiac trabeculation with gene mutation has not been reported previously. Here, we report a case of coexisting congenital left ventricular aneurysm and prominent left ventricular trabeculation in a patient with LIM domain binding 3 gene mutation.

**Case presentation:**

A 30-year-old Asian man showed paroxysmal sinus tachycardia and Q waves in an electrocardiogram health check. There were no specific findings in physical examinations and serological tests. A coronary-computed tomography angiography check showed normal coronary artery and no coronary stenosis. Both left ventricle contrast echocardiography and cardiac magnetic resonance showed rare patterns of a combination of an apical aneurysm-like out-pouching structure with a wide connection to the left ventricle and prominent left ventricular trabecular meshwork. High-throughput sequencing examinations showed a novel mutation in the *LDB3* gene (c.C793>T; p.Arg265Cys).

**Conclusions:**

Our finding indicates that the phenotypic expression of two heart conditions, congenital left ventricular aneurysm and prominent left ventricular trabeculation, although rare, can occur simultaneously with *LDB3* gene mutation. Congenital left ventricular aneurysm and prominent left ventricular trabeculation may share the same genetic background.

## Background

Congenital left ventricular aneurysm (LVA) is a rare cardiac malformation first described in 1816, and characterized as an akinetic or dyskinetic structure with a wide connection to the left ventricle [1]. Significant morbidity and mortality is associated with congenital LVA due to systemic embolization, valvular regurgitation, ventricular wall rupture, ventricular tachycardia, or sudden cardiac death [2]. The pathogenesis for congenital LVA during the complex embryologic development is not well understood, and several theories exist [3]. To date, no known genetic abnormalities have been found in this disease. Congenital LVA is associated with numerous other congenital anomalies [3], including those of the heart itself, or those of vascular or extracardiac structures; the most frequent associated cardiac abnormalities were ventricular septal defect [4], coronary anomalies [5], and atrial septal defect [6]. Abnormal cardiac trabeculation is observed in congenital heart diseases [7] and genetic cardiomyopathies [8, 9], and may serve as a measurable phenotypic marker that will allow insights into how genetic cardiomyopathies and congenital heart diseases arise and develop [10]. Gene mutations have been confirmed as the causative factors for genetic cardiomyopathies [11]. And there is increasing identification of genetic abnormalities linking the developmental defects in congenital heart diseases [12]. Therefore, whether gene mutation is associated with this rare combination is of interest. We report a rare case of coexisting congenital LVA and prominent left ventricular (LV) trabeculation with LIM domain binding 3 (*LDB3*) gene mutation (c.C793>T; p.Arg265Cys), which was not reported in the public databases of Human Gene Mutation Database (HGMD; http://www.hgmd.cf.ac.uk/ac/index.php) or Single Nucleotide Polymorphism database (dbSNP; http://www.ncbi.nlm.nih.gov/projects/SNP/). The genetic discovery from this case may open the door to a better understanding of abnormal cardiac development and affect clinical care of patients with congenital LVA.

## Case presentation

A 30-year-old Asian man was admitted to our hospital because of the finding of unusual Q waves of electrocardiogram (ECG) in his first health examination and an abnormal pattern of his left ventricle in a following transthoracic echocardiography check. He has no risk factors of cardiovascular diseases, and no history of coronary artery disease or myocarditis. He presented for years with unspecific symptoms like palpitation and vague, intermittent chest pain, which were unrelated to physical exertion, and he did not receive any medical intervention for these symptoms in the past.

**Fig. 1:**
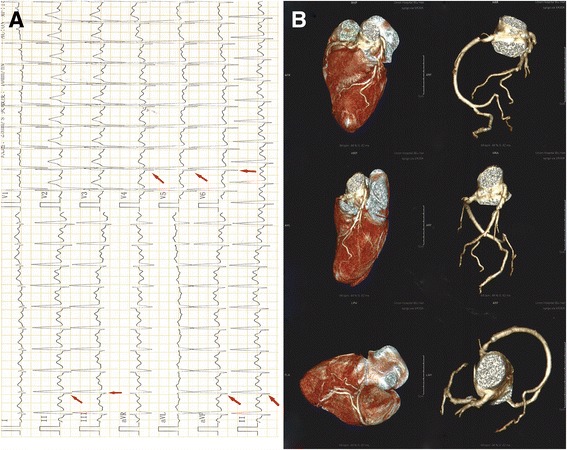
Electrocardiogram and computed tomography angiography at diagnosis. Panel **a** Twelve-lead electrocardiogram showing sinus tachycardia (121 beats per minute) and Q waves in I to III, avF, and V4 to V6 leads (*arrow*). Panel **b** Computed tomography angiography showing normal coronary artery and no coronary stenosis

**Fig. 2:**
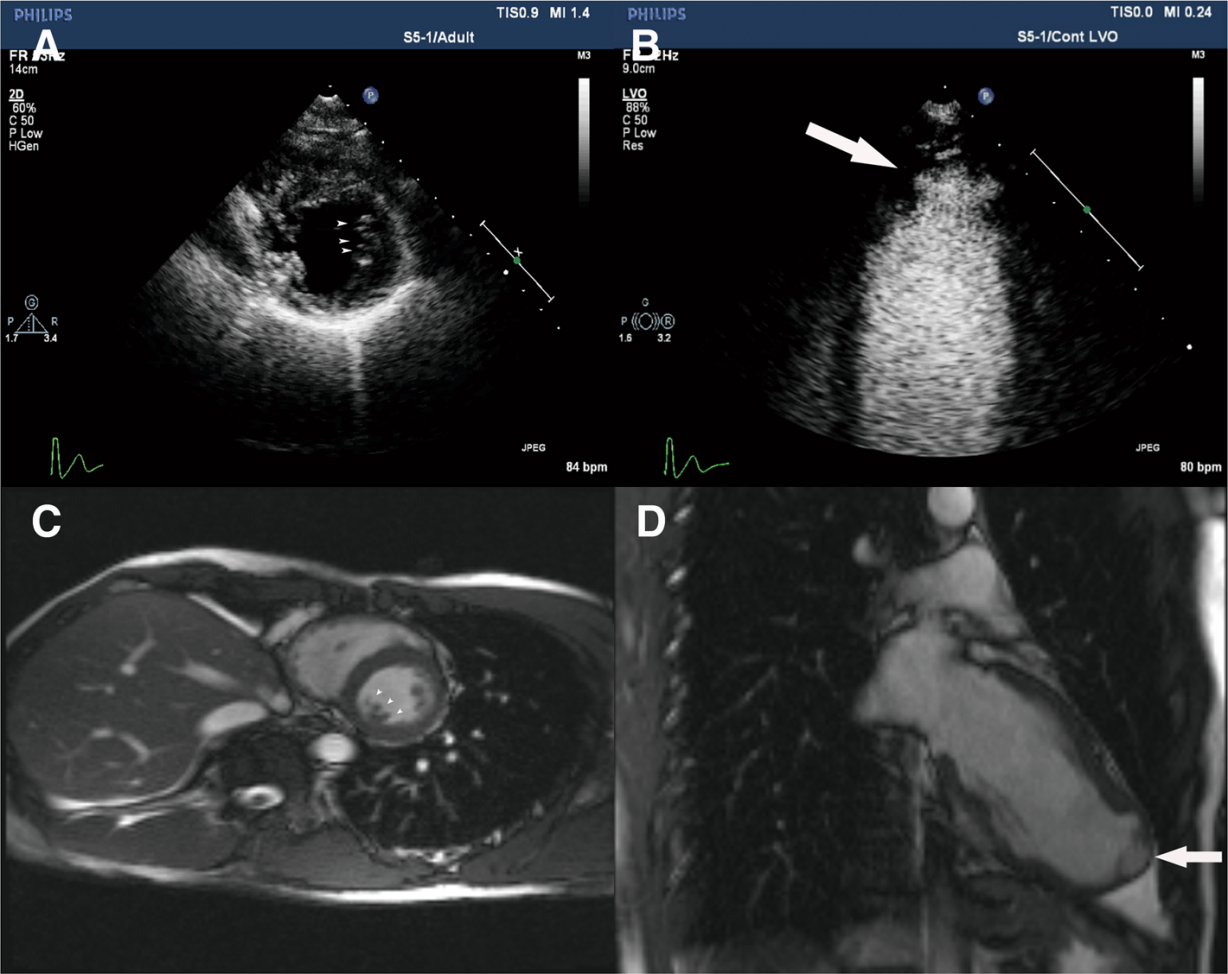
Contrast echocardiography and cardiac magnetic resonance at diagnosis. Panel **a**, **b** Contrast echocardiography. **a** Apical short-axis view of left ventricle showing prominent left ventricular trabeculae and deep intertrabecular recesses (*arrowheads*). **b** Transapical view of the left ventricular apex showing an aneurysm-like out-pouching structure with a wide connection to the left ventricle (*arrow*). Panel **c**, **d** Magnetic resonance imaging. **c** Left ventricle short-axis view showing prominent left ventricular trabeculae and deep intertrabecular recesses (*arrowheads*). **d** Left ventricular outflow tract view showing an apical protrusion with a wide connection to the left ventricle (*arrow*)

On general physical examination he had a body temperature of 36.7 °C and a heart rate of 84 beats per minute in a normal condition and 121 beats per minute in a cardiopalmus condition. His respiratory rate was 16 breaths per minute. He had a blood pressure of 110/72 mm Hg and an oxygen saturation of 98% on room air. His cardiac examination was normal; there were no murmurs or extracardiac sounds on auscultation. His complete physical examination including a neurological examination was unremarkable. Laboratory tests revealed: normal markers of myocardial injury, for example MB isoenzyme of creatine kinase (CK-MB), high-sensitive troponin I (hsTnI), lactate dehydrogenase (LDH), and aspartate aminotransferase (AST); a positive enterovirus (EVs) -ribonucleic acid (RNA); and negative coxsackievirus B (CoxB)3 -immunoglobulin M (IgM), CoxB5-IgM, and cytomegalovirus ©-IgM in the virologic examination. The antinuclear antibody (ANA) spectrum showed a positive anti-double-stranded deoxyribonucleic acid (dsDNA) antibody, and the titer of anti-ANA was within a normal range. Other ANAs were negative. The inflammatory indicators of C-reactive protein (CRP), antistreptolysin O (ASO), and erythrocyte sedimentation rate (ESR) were within the normal ranges. Routine laboratory tests for liver, renal, electrolytes, and blood glucose were normal. His low-density lipoprotein (LDL) cholesterol was mildly elevated (3.4 mmol/L) in the serum lipid profile and the other lipids were within normal range (Table 1). His blood, urine, and stool routine tests were all normal (data not shown). The ECG was reexamined and showed paroxysmal sinus tachycardia and Q waves in I-III, avF, and V4 to V6 leads (Fig. 1a). A subsequent coronary-computed tomography angiography (CTA) check showed normal coronary artery and no coronary stenosis (Fig. 1b). Both left ventricle contrast echocardiography and cardiac magnetic resonance (CMR) demonstrated that apical congenital LVA coexisted with prominent LV trabeculation (Fig. 2a–d). We re-evaluated his medical history carefully and comprehensively and found no family history of heart diseases or genetic diseases.

**Table 1:** Laboratory data of the patient

Parameters	Results	Reference values
Myocardial enzyme spectrum		
CK-MB (ng/ml)	0.5	<6.6
hsTnI (pg/ml)	2.1	<262
LDH (U/L)	135	109–245
AST (U/L)	16	8–40
Virologic test		
CoxB3-IgM	Negative	Negative
CoxB5-IgM	Negative	Negative
EVs-RNA	Positive	Negative
C-IgM	Negative	Negative
ANA spectrum		
Anti-ANA	<1:100	<1:100
SM	Negative	Negative
Anti-dsDNA	Positive	Negative
ACA (RU/ml)	5.0	<12
CENPB	Negative	Negative
nRNP	Negative	Negative
SSA	Negative	Negative
SSB	Negative	Negative
SCL-70	Negative	Negative
JO-1	Negative	Negative
RA-54	Negative	Negative
DM-53	Negative	Negative
D’E	Negative	Negative
Inflammatory indicators		
CRP (mg/L)	<3.28	<8
ASO (IU/ml)	<55.3	<200
ESR (mm/h)	2	<15
T-BIL (μmol/L)	12	5.1–19
D-BIL (μmol/L)	6.6	1.7–6.8
ALT (U/L)	15	5–40
ALP (U/L)	63	40–150
GGT (U/L)	21	11–50
A/G	1.9	1.5–2.5
LDL cholesterol (mmol/L)	3.4	2.7–3.1
Total cholesterol (mmol/L)	5.14	<5.2
HDL cholesterol (mmol/L)	1.38	1.16–1.42
Triglycerides (mmol/L)	0.88	<1.7
Fasting glucose (mmol/L)	4.61	3.9–6.1
HbA1C (%)	4.7	4.5–6.2
BUN (mmol/L)	3.9	2.9–8.2
Creatinine (μmol/L)	73.5	44–133
URIC (μmol/L)	408	208–428
CK (U/L)	116	38–174
LDH (U/L)	135	109–245
Na (mmol/L)	141	136–145
K (mmol/L)	4.0	3.5–5.2
Cl (mmol/L)	106	96–106
Ca (mmol/L)	2.32	2.03–2.54
CO_2_-CP (mmol/L)	24	22–28
P (mmol/L)	1.2	0.96–1.62
Mg (mmol/L)	0.75	0.70–1.10

*ACA* anticardiolipin antibody, *A/G* albumin/globulin, *ALP* alkaline phosphatase, *ALT* alanine transaminase, *ANA* antinuclear antibody, *Anti-dsDNA* anti-double-stranded DNA antibody, *ASO* antistreptolysin O, *AST* aspartate aminotransferase, *BUN* blood urea nitrogen, *C* cytomegalovirus, *Ca* calcium, *CENPB* centromere protein B, *CK* creatine kinase, *CK-MB,* MB isoenzyme of creatine kinase, *Cl* Chlorine, *CO*_*2*_ *-CP* carbon dioxide combining power, *CoxB* coxsackievirus B, *EVs* enterovirus, *C* cytomegalovirus, *CRP* C-reactive protein, *D-BIL* direct bilirubin, *D’E* anti-D’E polypeptide, *DM* dermatomyositis, *ESR* erythrocyte sedimentation rate, *EVs* enterovirus, *GGT* γ-glutamyl transpeptidase, *HbA1c* glycosylated hemoglobin, *HDL* high-density lipoprotein, *hsTnI* high-sensitive troponin I, *IgM* Immunoglobulin M, *Jo-1* anti-histidyl-transfer RNA synthetase *K* potassium, *LDH* lactate dehydrogenase, *LDL* low-density lipoprotein, *Mg* magnesium, *Na* sodium, *nRNP* nuclear ribonucleoprotein, *P* phosphorus, *RA* rheumatoid arthritis, *RNA* ribonucleic acid, *SCL* systemic sclerosis or scleroderma, *SM* Smith antibody, *SSA* Sjögren’s syndrome A, *SSB* Sjögren’s syndrome B, *T-BIL* total bilirubin, *URIC* uric acid

For further evaluating the genetic background of this rare combination, his DNA was isolated from a peripheral blood sample and screened for mutations by high-throughput sequencing, which aimed at cardiomyopathy-related genes, after informed consent was obtained. These mutations have been reported in five major cardiomyopathies, including hypertrophic cardiomyopathy (HCM), dilated cardiomyopathy (DCM), arrhythmogenic right ventricular cardiomyopathy (ARVC), restrictive cardiomyopathy (RCM), and LV non-compaction (LVNC) [11]. In this case, a heterozygous missense variant in *LDB3* gene (c.C793>T; p.Arg265Cys) was identified. The variant was considered to be probably pathogenic because of the following criteria: (1) it was not reported in the public databases of HGMD (http://www.hgmd.cf.ac.uk/ac/index.php) or dbSNP (http://www.ncbi.nlm.nih.gov/projects/SNP/); and (2) it predicted pathogenic mutation by multiple in silico algorithms (MutationTaster, PolyPhen-2, Align-GVGD, SIFT, and PANTHER). No other mutations were identified in the gene test. The final diagnosis of our patient was coexistence of congenital LVA and prominent LV trabeculation with *LDB3* mutation (c.C793>T; p.Arg265Cys). There was no occurrence of complications or new cardiac symptoms during a 3-month follow-up evaluation and he had a normal cardiac function after he was discharged. An annual follow-up was scheduled for further assessment.

## Discussion

To the best of our knowledge, this is the first report of a case of a combination of congenital LVA and prominent LV trabeculation with a gene mutation. Congenital LVA is a rare cardiac anomaly, which is described as an akinetic or dyskinetic structure with a wide connection to the left ventricle [3], and can be associated with ECG abnormalities and rhythm disturbances [13]. Human LV cardiac trabeculation is highly variable among individuals. Increased LV trabeculation is associated with other cardiac abnormalities, such as congenital heart diseases [7, 14] and cardiomyopathies, such as LVNC [15], HCM [8], and DCM [9], it has also been observed in healthy individuals [16–18]. Congenital LVA appears to be a developmental anomaly and was explained by a partial stop in the development of the embryologic ventricular wall, starting in the 4th embryonic week [3]. However, the underlying mechanisms of congenital LVA are still unclear.

The current diagnosis of congenital LVA is based on exclusion of other diseases which may induce acquired LV aneurysms, that is, coronary artery disease [19], autoimmune connective tissue disease [20], myocarditis [21], cardiomyopathies [22, 23], as well as traumatic causes [24]. Acquired aneurysms are very difficult to distinguish from congenital LV aneurysms without knowledge of the past history and a coronary angiogram. Most LV aneurysms are acquired aneurysms forming after myocardial infarction with systolic bulging of the scarred myocardium. This patient is absent of history of myocardial infarction. Laboratory tests revealed a normal myocardial enzyme spectrum, and a CTA check showed normal coronary artery and no coronary stenosis, which ruled out coronary artery disease. LV aneurysms may also result from myocarditis. Frustaci *et al*. [25] reported that among 353 patients with a diagnosis of myocarditis, 12 (3.3%) had single or multiple localized LV aneurysms [7]. This patient has neither history of myocarditis, nor clear evidence supporting the clinical diagnosis of myocarditis. His inflammatory indicators (CRP, ASO, and ESR) and markers of myocardial injury (for example, CK-MB or hsTnI) were all normal. Besides, virus serology is frequently used in clinical practice for the diagnosis of myocarditis [26]. We then conducted virological tests, aiming at the most commonly reported causative agents for myocarditis (that is, EVs, coxsackievirus, and C), and identified a positive EVs-RNA and negative serum antibodies for CoxB3-IgM, CoxB5-IgM, and C-IgM. A positive EVs-RNA suggests an EVs infection. However, EVs-RNA also commonly occurs in individuals with upper respiratory tract infection [27, 28], and even in healthy individuals [29, 30]. Moreover, recent research suggested that, for patients with suspected myocarditis, virus serology has no relevance for the diagnosis of myocardial infection [31]. For aiding precise diagnosis, we also used a CMR examination. CMR can distinguish between normal myocardial cells and those with myocarditis, providing a more accurate diagnosis of myocarditis [32]. This patient showed no features of edema, hyperemia and capillary leak, or necrosis and fibrosis, which are the three main kinds of cardiac tissue change seen in myocarditis. Therefore, the diagnosis of myocarditis cannot be made according to the diagnostic criteria in adults [33, 34]. Cardiomyopathies also have to be excluded since a right ventricular dysplasia can occasionally spread to the left ventricle [23], and apical LV aneurysms have also been described in the context of hypertrophic obstructive cardiomyopathy [22]. The diagnosis of HCM and right ventricular dysplasia relies on multiple imaging modalities, such as contrast-enhanced echocardiography and CMR [35, 36], and additional ECG markers also contribute to improve diagnostic sensitivity [36]. There was no image characterization in this patient that was compliant with the diagnostic criteria of these cardiomyopathies accessed by both contrast-enhanced echocardiography and CMR. What is more, a genetic study, aimed at cardiomyopathy-related genes [11], was also performed. And a mutation in the *LDB3* gene was identified. Autoimmune connective tissue disorder, such as systemic lupus erythematosus (SLE), has been reported to induce LV aneurysm [20]. Testing for ANAs is the screening test for patients in whom SLE is suspected [37]. We evaluated the ANA spectrum including 13 ANAs aimed at screening the main autoimmune connective tissue diseases, that is, SLE, Sjögren’s syndrome (SS), systemic scleroderma (SSc), dermatomyositis (DM), mixed connective tissue disease (MCTD), and rheumatoid arthritis (RA) [37, 38]. The ANA tests revealed a positive anti-dsDNA antibody and negative for other ANAs. Anti-dsDNA in ANA spectrum is a specific antibody for SLE, and the titer of anti-dsDNA tends to correlate with activity of disease [39]. However, the ANA test may produce a false-positive result, and ANAs are detected in 3 to 5% of healthy individuals or patients with other autoimmune or infectious diseases [39]. Hence, a positive anti-dsDNA alone is far from sufficient for diagnosis of SLE without systemic evaluation based on comprehensive clinical presentation, laboratory data, and other auxiliary examinations [39]. Traumatic cardiac aneurysm is irrelevant to this case because our patient has no history of trauma. Taken together, the clinical, echocardiographic, and imaging features of our case were in agreement with those described in the literature [3]. And the absence of clinical, laboratory, and imaging evidence of coronary artery disease, autoimmune connective tissue diseases, myocarditis, cardiomyopathies, or traumatic causes lends strong support to the diagnosis that the aneurysm occurred as a result of a congenital defect of the LV wall in the region of the LV apex.

We found a rare case combining both congenital LVA and prominent LV trabeculation in an adult. There have been concerns that excessive trabeculation may be a marker of underlying heart muscle disease [14]. For instance, LVNC is considered a distinct form of genetic cardiomyopathy in which the hallmark phenotypic feature is extensive LV trabeculation [15, 40]. Mutations in genes that encode various cardiac proteins have been identified as the cause of genetic cardiomyopathies [11]. And with recent advances in genomic technologies for more detailed evaluation of congenital heart diseases, genetic abnormalities linking the developmental defects in congenital heart disease have been increasingly identified [12]. Since congenital LVA presented with a typical phenotype in genetic cardiomyopathies, whether the same genetic background is shared within this rare combination is of interest. We then performed genetic tests aimed at five major genetic cardiomyopathies, including HCM, DCM, ARVC, RCM, and LVNC. The high-throughput sequencing tests showed a novel missense mutation located at c.C793>T in *LDB3* gene, which was submitted to HGMD and dbSNP databases after the detection.

The *LDB3* gene, also known as Z-band alternatively spliced PDZ motif (ZASP), encodes a PDZ-LIM domain-binding factor that plays an important role in maintaining the structural integrity of the striated muscle Z-disc in multiple species [41]. PDZ domain-containing proteins interact with each other in cytoskeletal assembly or with other proteins involved in targeting and clustering of membrane proteins. The ZASP protein is specifically expressed in heart and skeletal muscle [42]. Faulkner *et al*. [42] determined that the PDZ domain of ZASP binds to the COOH-terminal region of alpha-actinin-2 (ACTN2). Frey and Olson [43] showed that ZASP interacted strongly with three striated muscle-specific proteins (that is, calsarcin-1, calsarcin-2, and calsarcin-3). In addition, Lin *et al*. [41] found that the internal striated muscle ZASP-like motif (sZM) of the LDB3 protein interacted with the C terminus of human skeletal α-actin 1 (ACTA1), and exon 6 of *LDB3* alone was sufficient for interaction with ACTA1. The long ZASP isoform lacking exon 10 also interacted with ACTA1, indicating an additional actin-binding region encoded by the exon 8–11 junction that is not present in the other isoforms. These findings together suggested that *LDB3* gene is important for skeletal muscle structural integrity. Mutations in the *LDB3* gene have been identified in some cardiomyopathies, such as DCM, HCM, and LVNC [44–46]. In this case, we found the mutation in *LDB3* gene also presented with congenital LVA. Conceivably, the *LDB3* gene mutation may be associated with the development of myocardial lesion in this patient. Despite the specific genetic finding, it does not in itself change the current diagnostic and therapeutic strategy for congenital LVA; however, the identification of genetic abnormality, when integrated with the clinical characteristics, may influence the overall case assessment, and may appropriately impact the clinical recommendations in the setting of congenital LVA.

## Conclusions

This case presents the phenotypic expression of two heart conditions, congenital LVA and prominent LV trabeculation, coexisting with *LDB3* gene mutation, suggesting the same genetic background may be shared within congenital LVA and cardiomyopathies. However, a single case of such a rare combination with a single gene mutation does not strongly support the link. More evidence is still needed to elucidate the association of genetic variations and congenital LVA. Comprehensive diagnostic assessment may provide a better understanding of the genotype–phenotype correlation between these two heart conditions. Our finding may help cardiologists and medical scientists to gain new insights into the basic mechanisms leading to congenital LVA and abnormal cardiac trabeculation.

## Abbreviations

ACTA1: Alpha-actin 1
ACTN2: Alpha-actinin-2
ANA: Antinuclear antibody
ARVC: Arrhythmogenic right ventricular cardiomyopathy
ASO: Antistreptolysin O
AST: Aspartate aminotransferase
C: Cytomegalovirus
CK-MB: MB isoenzyme of creatine kinase
CMR: Cardiac magnetic resonance
CoxB: Coxsackievirus B
CRP: C-reactive protein
CTA: Computed tomography angiography
dbSNP: Single Nucleotide Polymorphism database
DCM: Dilated cardiomyopathy
DM: Dermatomyositis
dsDNA: Double-stranded DNA
ECG: Electrocardiogram
ESR: Erythrocyte sedimentation rate
EVs: Enterovirus
HCM: Hypertrophic cardiomyopathy
HGMD: Human Gene Mutation Database
hsTnI: High-sensitive troponin I
*LDB3*: LIM domain binding 3
LDH: Lactate dehydrogenase
LDL: Low-density lipoprotein
LV: Left ventricular
LVA: Left ventricular aneurysm
LVNC: Left ventricular non-compaction
MCTD: Mixed connective tissue disease
RA: Rheumatoid arthritis
RCM: Restrictive cardiomyopathy
SLE: Systemic lupus erythematosus
SS: Sjögren’s syndrome
SSC: Systemic scleroderma
sZM: Striated muscle ZASP-like motif
ZASP: Z-band alternatively spliced PDZ motif
